# Comparative analysis of the effects of two chest physical therapy interventions in patients with bronchiolitis during hospitalization period

**DOI:** 10.1590/S1679-45082014AO3230

**Published:** 2014

**Authors:** Renata Remondini, Adriana Zamprônio dos Santos, Giselle de Castro, Cristiane do Prado, Luiz Vicente Ribeiro Ferreira da Silva

**Affiliations:** 1 Hospital Israelita Albert Einstein, São Paulo, SP, Brasil.

**Keywords:** Bronchiolitis/rehabilitation, Physical therapy modalities, Child, hospitalized

## Abstract

**Objective:**

To evaluate and compare the effects of two chest physiotherapy interventions in patients hospitalized due to acute bronchiolitis.

**Methods:**

Prospective randomized study with a sample of 83 calls for 29 patients aged between 3 months and 1 year hospitalized for acute bronchiolitis. Patients were distributed randomly into two groups: Group 1, submitted to postural drainage, tapping and tracheal aspiration; and Group 2, submitted to postural drainage, expiratory acceleration flow and tracheal aspiration. Assessments were made before and 10 and 60 minutes after the end of therapy. Patients also underwent drug treatment. The endpoint was to compare two physical therapy interventions as to clinical improvement in infants with acute bronchiolitis by means of oxygen saturation and the Respiratory Distress Assessment Instrument score. The parents/guardians was requested to answer a questionnaire about the treatment applied before the last evaluation in order to measure their satisfaction related to the interventions made.

**Results:**

The groups were similar regarding the use of antibiotics and bronchodilators. A greater number of patients used corticosteroids in Group 2. A relevant improvement was observed on Respiratory Distress Assessment Instrument score with physical therapy, with reduction of the score 10 minutes after interventions, and the same score 60 minutes later, with no differences between techniques applied. There was no significant variation of pulse oximetry after chest physiotherapy. Most items assessed by the questionnaire had satisfactory answers.

**Conclusion:**

No differences were observed between groups regarding the items assessed (time required to discharge from study, pulse oximetry in room air and disease severity according to the Respiratory Distress Assessment Instrument score). Parents answered positively about the effects of therapy in the majority of items in the questionnaire, both for the expiratory acceleration flow technique and for tapping.

## INTRODUCTION

Acute bronchiolitis is considered one of the most common respiratory diseases in the first year of life. Respiratory syncytial virus (RSV) is the major etiological agent, but other viruses, such as parainfluenza, adenovirus, influenza A and B, may also cause bronchiolitis.^(1,2)^ These pathogens act upon ciliated epithelial cells, causing inflammation by producing inflammatory mediators.^(1,3)^ The clinical manifestations are nasal discharge, fever, cough, difficulty breathing and wheezing.^(4)^ The chest radiographic findings are characterized by hyperinflation, coarse infiltrates, atelectasis and peribronchial cuffing.^(1)^

The treatment of bronchiolitis is quite controversial and includes hydration, oxygenation, respiratory therapy, and medications, including bronchodilators, epinephrine, mucolytics, and inhaled corticosteroids.^(1,5,6)^ The use of supplemental oxygen is indicated in patients with peripheral oxygen saturation (SpO_2_)<92%.^(1)^ Chest physiotherapy has been used with the objective of bronchial clearance and lung deflation and alveolar recruitment through various techniques such as postural drainage, expiratory acceleration flow (EAF), vibrocompression, tapping and airway aspiration.^(7,8)^

Postural drainage consists in positioning the patient so as to use the force of gravity to help enhance the drainage of mucus from lobes and specific segments of the lungs into the central airways.^(9)^ Tapping aims to mobilize and remove lung secretions, facilitating their drainage from peripheral to central regions, and promoting the elimination of these secretions, therefore improving lung function.^(10)^ Tracheal suction is considered an effective technique for tracheobronchial clearance in children with bronchiolitis, taking into account that approximately 60% of the respiratory resistance is located in the upper airway. The aspiration technique should be a sterile procedure for secretion removal using a vacuum system.^(1)^

Some of the physiotherapy techniques used in pediatric patients are an adaptation of methods used in adult patients, such as tapping, vibrocompression and postural drainage. Over the years, specific techniques have been developed for each age group, highlighting among them the EAF, which consists of a passive stimulation in the thoracic region of the patient with prolonged expiratory time. The acceleration pressure applied by the hand of the therapist must be symmetric. The expiratory pressure should be started as the patient begins to exhale. The acceleration must be performed at a speed higher than the normal expiration rate and near the coughing speed. One of the hands of the physiotherapist is positioned in the chest (expiratory pressure hand), and the other hand in the lower ribs, minimizing the increase in abdominal pressure.^(10)^

The choice of a particular physical therapy maneuver often depends on the preference of the therapist, the type of training and the protocols of each service.^(10,11)^ Further studies must be conducted to evaluate the effectiveness of various respiratory physiotherapy techniques in patients with acute bronchiolitis.

## OBJECTIVE

To evaluate and compare the effects of two physical therapy interventions after 10 and 60 minutes of treatment in patients with acute bronchiolitis during hospitalization; to assess the time required for a patient to be discharged from the study, comparing the two groups, by evaluating pulse oximetry on room air (RA) and disease severity score using the Respiratory Distress Assessment Instrument (RDAI) score system; to evaluate the parental acceptance of the physical therapy interventions.

## METHODS

This study was approved by the Research Ethics Committee of the *Hospital Israelita Albert Einstein*, under the approval number 10/1352.

This was a prospective randomized study in which we evaluated 83 visits of 29 patients with acute bronchiolitis hospitalized in the intensive care unit (ICU) and the pediatric ward of the *Hospital Israelita Albert Einstein*, from July 2010 to December 2011.

The study included children aged three months to one year with clinical diagnosis of acute bronchiolitis, after the parents or guardians signed an Informed Consent Form. No children with uncorrected congenital heart disease, neuropathy, underlying lung disease, indication for ventilatory support, RDAI score ≤4 associated to SpO_2_≥92% on RA, or without parent or guardian agreement to participate in the research were included. Only four patients were excluded from the study, due to refusal of the parents, for non-acceptance of the EAF maneuver.

In this study, patients were randomized into two groups: Group 1 underwent postural drainage associated with tapping and tracheal aspiration; and Group 2 underwent postural drainage associated with EAF and tracheal aspiration. In Group 1, 16 patients were included, totaling 48 treatment sessions, and in Group 2, 13 patients were included, totaling 35 treatment sessions. These interventions were performed in all cases, until discharge from the study or from the hospital.

The eligible subjects were assessed daily, on the first physiotherapy visit of the day, at three different moments (before, 10 minutes after, and 60 minutes after the physical therapy intervention), by the same therapist. All physiotherapists in the Maternal and Child Health Department were trained to conduct the study.

We considered the patient apt to be discharged from the study, that is completed data collection, when the patient presented a lower disease severity score (RDAI score ≤4) associated with adequate oxygenation on RA (SpO_2_≥92%).

Before the last physical therapy intervention, a questionnaire consisting of 14 questions was applied to the parents or guardians to evaluate their satisfaction regarding the physical therapy interventions. The parents or guardians were informed about the treatment and were present in all cases, observing the clinical course of the child.

The endpoint was to compare two physical therapy interventions as to clinical improvement in infants with acute bronchiolitis, by means of SpO_2_ and the RDAI score.

The RDAI score assessed the disease severity by analyzing clinical variables, with scores 1-17, according to the location and intensity of retractions and wheezing. The score was considered mild when the value was ≤4.^(12,13)^ Pulse oximetry on RA was measured using a Nellcor^®^, OxiMax^®^ N560 pulse oximeter, with the patient deprived from supplemental oxygen for a period between 5 and 10 minutes. Patients with values of pulse oximetry below 90% immediately resumed the use of oxygen, and pulse oximetry values were categorized into<92% and ≥92%.

Postural drainage was performed in the supine, lateral (left and right) and/or sitting positions. EAF and tapping were performed for 10 minutes in each position.

The aspiration was a sterile procedure, lasting 10-15 seconds, with the use of a tracheal aspiration tube and surgical gloves. Before the procedure, a 0.9% saline solution was instilled into the patient’s nostrils for upper airway humidification.

The number of treatment sessions for each patient was determined by the physical therapist as needed, according to the disease severity, varying from one to four daily sessions, similar for both groups.

For statistical analysis, a comparison of the characteristics in the groups was performed using Pearson’s χ^2^ tests and Fisher exact tests. For time-course analyses, we used the Friedman’s test to evaluate the difference between the different moments, and the Mann-Whitney test to assess the difference between the groups in the case of non-normally distributed variables. For normal distribution variables, we used analysis of variance with repeated measures, evaluating the presence of an interaction between group and time. In the presence of an interaction, a Bonferroni correction to the significance level (adjusted significance level of 0.05/number of comparisons) was applied. For all tests in the study a significance level of 5% was taken into account. The analyses were performed with the aid of the Statistical Package for the Social Science (SPSS Inc. Released 2008, SPSS Statistics for Windows, Version 17.0. Chicago: SPSS Inc.).

## RESULTS

The groups were similar with respect to gender, age, use of antibiotics and bronchodilators. There was a higher number of visits to ICU in Group 1, and a larger number of visits of patients using corticosteroids in Group 2 (Table 1).

**Table 1:** Characteristics of the groups

Characteristics	Group 1 n (%)	Group 2 n (%)	p value
Gender			
Male	34 (70.8)	28 (80.0)	0.343
Female	14 (29.2)	7 (20.0)	
Age in months (average)	5.47	6.28	
Unit			
Ward	39 (81.3)	34 (97.1)	0.028*
ICU	9 (18.8)	1 (2.9)	
Bronchodilator			
Yes	48 (100.0)	35 (100.0)	
Antibiotic			
No	31 (64.6)	19 (54.3)	0.344
Yes	17 (35.4)	16 (45.7)	
Corticosteroid			
No	29 (60.4)	13 (37.1)	0.036*
Yes	19 (39.6)	22 (62.9)	

Pearson’s χ^2^ tests and Fisher exact tests to compare the characteristics in the groups.* Analysis of variance with repeated measures for variables with normal distribution (interaction between group and time); Bonferroni correction to the significance level (adjusted significance level =0.05/number of comparisons). ICU: intensive care unit.

The median time to discharge in Group 1 of the study was 3 days, ranging from 2 to 5 days. In Group 2, the median time to discharge was 2 days, ranging from 1 to 5 days. There was no statistically significant difference between the two groups (p=0.408).

In most visits, the patients had SpO_2_≥92%, without significant variations over the course of time for both groups (Table 2).

**Table 2:** Comparison of clinical signs over the course of time in the two groups

Clinical signs	Group 1 (n=48)				Group 2 (n=35)			
	Before (%)	10 minutes (%)	60 minutes (%)	p value (time)	Before (%)	10 minutes (%)	60 minutes (%)	p value (time)
Pulse oximetry on room air (%)								
<92	6 (12.5)	2 (4.2)	5 (10.4)	0.307	7 (20)	3 (8.6)	3 (8.6)	0.234
≥92%	42 (87.5)	46 (95.8)	43 (89.6)		28 (80)	32 (91.4)	32 (91.4)	
p value (groups)					0.356	0.408	0.780	
RDAI								
Mean (standard deviation)	5.02 (2.07)	3.06 (1.77)	3.13 (1.81)	<0.001	5.8 (1.51)	3.6 (1.82)	3.26 (1.96)	<0.001*
p value (groups)					0.196			


Friedman’s test to assess the difference between the different moments; Mann-Whitney test to evaluate the difference between the groups in the case of non-normal distributed variables.* Analysis of variance with repeated measures for variables with normal distribution (interaction between group and time); Bonferroni correction to the significance level (adjusted significance level = 0.05/number of comparisons). RDAI: Respiratory Distress Assessment Instrument.

RDAI: Respiratory Distress Assessment Instrument; PD: postural drainage; TP: tapping; NTA: nasotracheal aspiration; EAF: expiratory acceleration flow.

**Figure 1:**
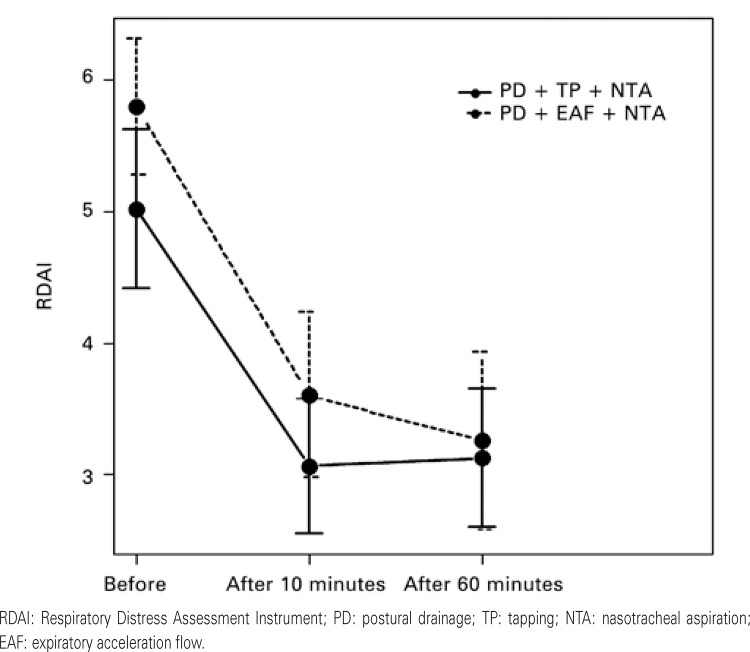
Confidence intervals of 95% for the mean Respiratory Distress Assessment Instrument scores, according to group and time of assessment

Time-course RDAI comparisons showed that the two groups behaved similarly (p=0.098) and there were no differences between the two groups (p=0.196). Both groups showed a RDAI decrease 10 minutes after the intervention (p<0.001) and maintained these values after 60 minutes. In Group 1, the score went from 5.02 to 3.06 after 10 minutes and to 3.13 after 60 minutes. In the Group 2, the score went from 5.8 to 3.6 after 10 minutes and to 3.26 after 60 minutes (Figure 1 and Table 2).

The answers on the effects of the physiotherapy were satisfactory in the majority of items assessed by the questionnaire that was used, with no difference between the two groups (Table 3).

**Table 3:** Questionnaire items on the parental/guardian satisfaction regarding the physical therapy interventions conducted

Questionnaire item	Group 1 n (%)	Group 2 n (%)	p value
Child was calm during physiotherapy maneuvers			
No	1 (6.3)	0	>0.99
Yes	15 (93.8)	13 (100.0)	
Classification of physiotherapy maneuvers			
Relaxing	15 (100.0)	13 (100.0)	
Tiring	0	0	
Physiotherapy maneuvers have helped to improve the child's breathing			
No	1 (6.3)	0	>0.99
Yes	15 (93.8)	13 (100.0)	
Child was calm during nasotracheal aspiration			
No	16 (100.0)	13 (100.0)	
Yes	0	0	
Tracheal aspiration helped improve the child's breathing			
No	0	0	
Yes	16 (100.0)	13 (100.0)	
Physiotherapy treatments performed by different professionals were similar			
No	0	0	
Yes	16 (100.0)	13 (100.0)	
Child benefited from the physiotherapy treatment			
No	1 (6.3)	0	>0.99
Yes	15 (93.8)	13 (100.0)	
Improved sleep			
No	3 (18.8)	3 (23.1)	>0.99
Yes	13 (81.3)	10 (76.9)	
Improvement in difficulty breathing			
No	0	1 (7.7)	0.448
Yes	16 (100.0)	12 (92.3)	
Improved feeding			
No	9 (56.3)	6 (46.2)	0.588
Yes	7 (43.8)	7 (53.8)	
Improvement in nasal congestion			
No	2 (12.5)	2 (15.4)	>0.99
Yes	14 (87.5)	11 (84.6)	
Improved mood			
No	9 (56.3)	5 (38.5)	0.340
Yes	7 (43.8)	8 (61.5)	
Conducting researches like this one is important			
No	0	0	
Yes	16 (100.0)	13 (100.0)	
Would recommend chest physiotherapy for a relative or friend			
No	1 (6.3)	0	>0.99
Yes	15 (93.8)	13 (100.0)	

Analysis of variance with repeated measures for variables with normal distribution (interaction between group and time); Bonferroni correction to the significance level (adjusted significance level =0.05/number of comparisons).

## DISCUSSION

The treatment of bronchiolitis usually consists of pharmacological therapy, which aims to attenuate the pathophysiological condition, and physical therapy, with the objective of providing bronchial clearance, optimization of lung re-expansion and improved respiratory mechanics, with a consequent prevention of pulmonary complications.^(14,15)^

Although the pathophysiology of RSV infection suggests the effectiveness of the use of corticosteroids with anti-inflammatory action in the treatment of bronchiolitis, studies show no benefit from the use of this therapy.^(16)^ According to the Clinical Guidelines on Complementary Health of the Brazilian Society of Pediatrics, corticosteroids are not recommended in the outpatient or inpatient treatment of acute bronchiolitis (recommendation grade A).^(17)^

The use of bronchodilators is more effective in the early stage of the infection, when the small airways are not obstructed with secretions.^(16)^ According to the Clinical Guidelines on Complementary Health of the Brazilian Society of Pediatrics, the routine use of inhaled bronchodilators is not recommended in the outpatient or inpatient treatment to improve the prognosis of acute bronchiolitis (recommendation grade A).^(17)^

In the study by Pinto et al., approximately 40% of patients used antibiotics, with no differences between groups, similarly to a recent randomized study, with 184 children younger than 12 months of age, hospitalized with bronchiolitis. Antibiotic treatment did not reduce the duration of hospitalization or the need for supplemental oxygen, leading to the conclusion that, in bronchiolitis, treatment with antibiotics should not be used routinely, but only when there is evidence of a bacterial infection.^(16)^

Respiratory therapy has been used routinely in patients with bronchiolitis, but its benefits are still questioned by the lack of clinical trials on the topic and the limited methodological quality of the researches, which undermines any claim that may be made regarding the positive effects of physiotherapy in patients with bronchiolitis.^(18)^

In a recent study, in which physical therapy was performed in all patients, less need for ICU admission and ventilatory support has been observed, compared to previously reported data.^(14)^

Some studies reported that physical therapy should not be indicated in acute bronchiolitis, because the bronchial clearance maneuvers may cause agitation in the child, leading to hypoxemia and triggering bronchospasm. On the other hand, studies have reported that physical therapy cause great benefit in these children, promoting decreased hospital stay and avoiding the need for ventilatory support.^(6,19)^

The literature also compares different interventions, such as tapping and vibration, associated with postural drainage, and does not indicate benefits in the clinical course of the disease.^(8,20)^ However, French studies demonstrated benefits in removing secretion by the EAF technique, besides the interventions mentioned above.^(21)^ EAF potentiates normal lung physiology through changes in airflows, promoting bronchial clearance and homogenization of pulmonary ventilation.^(22)^

In the study by Castro et al., which evaluated the effects of chest physiotherapy in patients with bronchiolitis, no statistically significant differences were observed in relation to oxygen therapy and SpO_2_ before and after the treatments.^(23)^ In this study, in both groups, in the majority of cases, the patients had SpO_2_≥92% at all observed times, without significant changes.

The RDAI score shows good reliability in assessing the presence and location of wheezing and retractions, with scores 1-17, according to the intensity of the signs. Higher scores indicate more severe disease, therefore a score <4 indicates mild disease, and a score >15 indicates severe disease.^(13)^ In this study, both groups behaved similarly in the RDAI score assessment, with a significant reduction from the score “before” to the “after 10 minutes”.

During the analysis of the questionnaire applied to parents/guardians, an immediate reduction of respiratory distress was observed after chest physiotherapy, and this benefit was evident. Despite the lack of scientific evidence about the effects of chest physiotherapy in patients with acute bronchiolitis, bronchial clearance techniques have been requested to treat these patients in several centers, due to improved respiratory symptoms and reduced pulmonary complications observed in clinical practice. These techniques are recommended in cases of bronchial obstruction due to secretions.^ (18)^

In the daily application of physical therapy techniques, we identified a more rapid reduction of respiratory and clinical symptoms, such as decreased fever and dyspnea, increased appetite, improved pulmonary auscultation and cough.^(24)^ In this study, satisfactory responses were observed regarding the effects of physiotherapy on most items evaluated by parents/guardians. All patients improved breathing after the physical therapy intervention. A small difference was observed between patients who did or did not show improvement in feeding and mood, but without statistical significance.

Among the limitations of this study, we mention the dissimilarity of the groups regarding the use of corticosteroids and ICU stay, and refusals by parents in relation to the EAF maneuver due to agitation and irritability observed in children undergoing this maneuver.

## CONCLUSION

No differences were observed between the groups regarding the items measured (time to patient discharge from the study, pulse oximetry on room air, and the disease severity score by Respiratory Distress Assessment Instrument score system). Chest physiotherapy promoted significant improvement in respiratory distress in patients with bronchiolitis, mainly after 10 minutes, maintaining adequate oxygenation at all times, similarly in both groups. Moreover, satisfactory answers were observed from parents/guardians about the effects of physiotherapy on most items assessed, both for expiratory flow acceleration technique and tapping. Despite the positive results of this study, additional clinical trials must be conducted in order to evaluate and compare the effectiveness of different physiotherapy techniques in these patients.
