# Supplementary material for: Comparative analysis of the effects of two chest physical therapy interventions in patients with bronchiolitis during hospitalization period
Source: Einstein (Sao Paulo). 2014 Oct-Dec;12(4):452–8. doi: 10.1590/S1679-45082014AO3230 (PMC4879911; doi:10.1590/S1679-45082014AO3230)
Supplement: Translation

# Translation: Análise comparativa dos efeitos de duas intervenções de fisioterapia respiratória em pacientes com bronquiolite durante o período de internação hospitalar

**Authors:** Renata Remondini, Adriana Zamprônio dos Santos, Giselle de Castro, Cristiane do Prado, Luiz Vicente Ribeiro Ferreira da Silva

**Affiliations:** 1 Hospital Israelita Albert Einstein, São Paulo, SP, Brasil.

**Keywords:** Bronquiolite/reabilitação, Modalidades de fisioterapia, Criança hospitalizada

## Abstract

**Objetivo:**

Avaliar e comparar os efeitos de duas intervenções fisioterapêuticas em pacientes com bronquiolite aguda durante internação hospitalar.

**Métodos:**

Estudo prospectivo e randomizado, com amostra de 83 atendimentos de 29 pacientes com idade entre 3 meses e 1 ano internados por bronquiolite aguda. Os pacientes foram randomizados em dois grupos: Grupo 1, submetido à drenagem postural, tapotagem e aspiração nasotraqueal; e Grupo 2, submetido à drenagem postural, aceleração do fluxo expiratório e aspiração nasotraqueal. Foram realizadas avaliações antes e 10 e 60 minutos após o término da fisioterapia. Os pacientes também foram submetidos a tratamento medicamentoso. O objetivo foi comparar duas intervenções fisioterapêuticas quanto à melhora clínica de lactentes com bronquiolite aguda por meio da saturação periférica de oxigênio e do escore *Respiratory Distress Assessment* *Instrument*. Um questionário sobre o tratamento ministrado foi aplicado ao responsável, antes da última avaliação, para mensurar a satisfação dos mesmos em relação às intervenções.

**Resultados:**

Os grupos foram similares em relação ao uso de antibiótico e broncodilatador. Observamos um número maior de pacientes que utilizaram corticoide no Grupo 2. Observou-se melhora significante no escore *Respiratory Distress Assessment Instrument* com o tratamento fisioterapêutico, com redução do mesmo, 10 minutos após as intervenções e manutenção dos valores 60 minutos após, sem diferenças entre as técnicas empregadas. Não houve variação significativa da oximetria de pulso após o atendimento fisioterapêutico. A maioria dos itens avaliados pelo questionário teve respostas satisfatórias.

**Conclusão:**

Não foram observadas diferenças entre os grupos em relação aos itens avaliados (tempo necessário para o paciente atingir alta do estudo, oximetria de pulso em ar ambiente e gravidade da doença pelo escore *Respiratory Distress Assessment Instrument*). O questionário teve respostas favoráveis dos pais sobre efeitos da fisioterapia na maioria dos itens, tanto para a técnica aceleração do fluxo expiratório quanto para a tapotagem.

## INTRODUÇÃO

A bronquiolite aguda é considerada uma das doenças respiratórias mais comuns no primeiro ano de vida. O vírus sincicial respiratório (VSR) é o principal agente etiológico, contudo outros vírus, como parainfluenza, adenovírus, influenza A e B, também podem ocasionar bronquiolite.^(1,2)^ Esses patógenos agem nas células epiteliais ciliadas, ocasionando inflamação pela produção de mediadores inflamatórios.^(1,3)^ As manifestações clínicas são coriza, febre, tosse, dificuldade respiratória e chiado.^(4)^ Os achados radiográficos de tórax são caracterizados por hiperinsuflação, infiltrados grosseiros, atelectasias e preenchimento peribrônquico.^(1)^

O tratamento da bronquiolite é bastante controverso e inclui hidratação, oxigenação, fisioterapia respiratória e medicamentos, como broncodilatadores, adrenalina, mucolíticos e corticoide inalatório.^(1,5,6)^ É indicado oxigênio suplementar em pacientes com saturação periférica de oxigênio (SpO_2_) <92%.^(1)^ A fisioterapia respiratória tem sido utilizada com os objetivos de desobstrução brônquica, desinsuflação pulmonar e recrutamento alveolar, por meio de diversas técnicas, como drenagem postural, aceleração do fluxo expiratório (AFE), vibrocompressão, tapotagem e aspiração das vias aéreas.^(7,8)^

A drenagem postural consiste no posicionamento do paciente, favorecendo a aplicação de forças gravitacionais, que aumentam o transporte de muco de lobos e segmentos específicos do pulmão, em direção às vias aéreas centrais.^(9)^ A tapotagem tem como objetivo mobilizar e remover a secreção pulmonar, facilitando sua condução das regiões periféricas para as centrais, e promovendo sua eliminação, melhorando a função pulmonar.^(10)^ A aspiração nasotraqueal é considerada uma técnica efetiva para a desobstrução traqueobrônquica de crianças com bronquiolite, levando-se em consideração que aproximadamente 60% da resistência respiratória está localizada nas vias aéreas superiores. A técnica de aspiração deve ser um procedimento estéril para remoção de secreção por meio de um sistema a vácuo.^(1)^

Algumas técnicas de fisioterapia utilizadas na faixa etária pediátrica foram uma adaptação dos métodos utilizados em pacientes adultos, como tapotagem, vibrocompressão e drenagem postural. No decorrer dos anos, surgiram técnicas específicas para cada faixa etária, destacando-se, entre elas, a AFE, que consiste na AFE por meio de um estímulo passivo na região torácica do paciente, com prolongamento do tempo expiratório. A pressão de aceleração dada pela mão do fisioterapeuta deve ser simétrica. O início da pressão expiratória se dá assim que o paciente inicia a expiração. A aceleração deve ser feita a uma velocidade superior à da expiração normal e próxima à da tosse. As mãos do fisioterapeuta se posicionam uma no tórax (mão da pressão expiratória) e a outra nas costelas inferiores, minimizando o aumento da pressão abdominal.^(10)^

A escolha de uma determinada manobra fisioterapêutica depende, muitas vezes, da preferência do profissional, do tipo de formação e dos protocolos de cada serviço.^(10,11)^ É necessária a realização de mais estudos que avaliem a efetividade de diferentes técnicas de fisioterapia respiratória em pacientes com bronquiolite aguda.

## OBJETIVO

Avaliar e comparar os efeitos de duas intervenções fisioterapêuticas após 10 e 60 minutos da terapia em pacientes com bronquiolite aguda durante a internação hospitalar; comparar o tempo necessário para o paciente atingir alta do estudo por meio da avaliação da oximetria de pulso em ar ambiente (AA) e gravidade da doença pelo escore *Respiratory Distress Assessment Instrument* (RDAI) entre os dois grupos; avaliar a aceitação dos pais em relação às intervenções fisioterapêuticas.

## MÉTODOS

Este estudo foi aprovado pelo Comitê de Ética em Pesquisa do Hospital Israelita Albert Einstein, sob o número de aprovação 10/1352.

Estudo prospectivo e randomizado, no qual foram avaliados 83 atendimentos de 29 pacientes com bronquiolite aguda internados na unidade de terapia intensiva (UTI) e ala pediátrica do Hospital Israelita Albert Einstein, no período de julho de 2010 a dezembro de 2011.

Foram incluídas no estudo crianças com idade de três meses a um ano com diagnóstico clínico de bronquiolite aguda, após assinatura do Termo de Consentimento Livre e Esclarecido pelos pais ou responsáveis. Não foram incluídas crianças com cardiopatia congênita não corrigida, neuropatia, doença pulmonar de base, necessidade de suporte ventilatório, escore clínico de desconforto respiratório RDAI ≤4 associado à SpO_2_≥92% em AA e não concordância dos responsáveis em participar da pesquisa. Apenas quatro pacientes não foram incluídos no estudo, devido à recusa dos pais por não aceitarem a manobra de AFE.

Neste estudo, os pacientes foram randomizados em dois grupos: Grupo 1, submetido à drenagem postural associada à tapotagem e à aspiração nasotraqueal; e Grupo 2, submetido à drenagem postural associada à AFE e à aspiração nasotraqueal. No Grupo 1, foram incluídos 16 pacientes, totalizando 48 atendimentos e, no Grupo 2, 13 pacientes, totalizando 35 atendimentos. Essas intervenções foram realizadas em todos os atendimentos, até a alta do estudo ou alta hospitalar.

As crianças elegíveis ao estudo foram avaliadas diariamente, no primeiro atendimento fisioterapêutico do dia, em três diferentes momentos (antes, 10 minutos e 60 minutos após a intervenção fisioterapêutica), pelo mesmo profissional. Todas as fisioterapeutas do Departamento Materno-Infantil foram treinadas para a realização do estudo.

Consideramos que o paciente estava apto a receber alta do estudo, ou seja, finalização da coleta de dados, quando apresentasse menor gravidade da doença (escore RDAI≤4), associada à oxigenação adequada em AA (SpO_2_≥92%).

Antes da última intervenção fisioterapêutica foi entregue aos pais ou responsável um questionário composto por 14 questões, para mensurar a satisfação dos mesmos em relação às intervenções fisioterapêuticas realizadas. Os pais ou responsáveis foram orientados quanto ao tratamento e estiveram presente em todos os atendimentos, observando a evolução clínica da criança.

O objetivo foi comparar duas intervenções fisioterapêuticas quanto a melhora clínica de lactentes com bronquiolite aguda, por meio da SpO_2_ e do escore RDAI.

O escore RDAI avalia a gravidade da doença analisando variáveis clínicas, com pontuação de 1 a 17, de acordo com a localização e a intensidade de retrações e sibilos. Esse escore foi considerado leve quando o valor era ≤4.^(12,13)^ A oximetria de pulso em AA foi verificada por meio de oxímetro de pulso da marca Nellcor^®^, modelo OxiMax^®^ N560, estando o paciente sem oxigênio suplementar por um período entre 5 e 10 minutos. Pacientes com valores de oximetria de pulso abaixo de 90% retomavam o uso do oxigênio de imediato, e os valores de oximetria de pulso foram categorizados em <92% e ≥92%.

A drenagem postural foi realizada nos decúbitos dorsal, laterais (direito e esquerdo) e/ou posição sentada. A tapotagem e a AFE foram realizadas durante 10 minutos em cada postura.

A aspiração consistiu de procedimento estéril, com duração de 10 a 15 segundos, utilizando sonda de aspiração traqueal e luva cirúrgica. Antes do procedimento, foi instilado soro fisiológico 0,9% nas narinas para a umidificação das vias aéreas superiores.

O número de atendimentos de cada paciente foi determinado pela fisioterapeuta, conforme a necessidade e a gravidade, com variabilidade de uma a quatro sessões ao dia, semelhante para os dois grupos.

Na análise estatística, as comparações das características nos grupos foram realizadas com testes de χ^2^ de Pearson ou exatos de Fisher. Para as análises ao longo do tempo, foram utilizados testes de Friedman, para avaliar a diferença entre os diferentes momentos, e testes de Mann-Whitney, para avaliar a diferença entre os grupos, no caso das variáveis com distribuição não normal. Para as variáveis com distribuição normal, utilizamos análise de variância com medidas repetidas, estudando a presença de interação entre grupo e tempo. Na presença de interação, foram realizadas correções de Bonferroni ao nível de significância (nível de significância ajustado de 0,05/número de comparações). Para todos os testes do estudo foi considerado um nível de significância de 5%. As análises foram realizadas com o auxílio do programa estatístico *Statistical Package for the Social Science* (SPSS Inc. Released 2008, SPSS *Statistics for Windows, Version* 17.0. Chicago: SPSS Inc.).

## RESULTADOS

Os grupos foram similares em relação a sexo, idade, uso de antibiótico e broncodilatador. Houve maior número de atendimentos em UTI no Grupo 1 e um número maior de atendimentos referentes a pacientes que utilizaram corticoide no Grupo 2 (Tabela 1).

**Tabela 1:** Características dos grupos

Características	Grupo 1 n (%)	Grupo 2 n (%)	Valor de p
Gênero			
Masculino	34 (70,8)	28 (80,0)	0,343
Feminino	14 (29,2)	7 (20,0)	
Idade em meses (média)	5,47	6,28	
Local			
Ala	39 (81,3)	34 (97,1)	0,028*
UTI	9 (18,8)	1 (2,9)	
Broncodilatador			
Sim	48 (100,0)	35 (100,0)	
Antibiótico			
Não	31 (64,6)	19 (54,3)	0,344
Sim	17 (35,4)	16 (45,7)	
Corticoide			
Não	29 (60,4)	13 (37,1)	0,036*
Sim	19 (39,6)	22 (62,9)	

Testes de χ^2^ de Pearson e exatos de Fisher para comparações das características nos grupos. * Análise de variância com medidas repetidas para variáveis com distribuição normal (interação entre grupo e tempo); correções de Bonferroni ao nível de significância (nível de significância ajustado =0,05/número de comparações). UTI: unidade de terapia intensiva.

O tempo mediano até alta do estudo no Grupo 1 foi de 3 dias, variando de 2 a 5 dias. No Grupo 2, o tempo mediano até alta foi de 2 dias, variando de 1 a 5 dias. Não houve diferença estatisticamente significante entre os dois grupos (p=0,408).

Na maioria dos atendimentos os pacientes apresentaram SpO_2_≥92%, sem grandes variações ao longo do tempo para os dois grupos (Tabela 2).

**Tabela 2:** Comparação dos sinais clínicos ao longo do tempo nos dois grupos

Sinais clínicos	Grupo 1 (n=48)				Grupo 2 (n=35)			
	Pré (%)	10 minutos (%)	60 minutos (%)	Valor de p (tempo)	Pré (%)	10 minutos (%)	60 minutos (%)	Valor de p (tempo)
Oximetria de pulso em ar ambiente (%)								
<92	6 (12,5)	2 (4,2)	5 (10,4)	0,307	7 (20)	3 (8,6)	3 (8,6)	0,234
≥92%	42 (87,5)	46 (95,8)	43 (89,6)		28 (80)	32 (91,4)	32 (91,4)	
Valor de p (grupos)					0,356	0,408	0,780	
RDAI								
Média (desvio padrão)	5,02 (2,07)	3,06 (1,77)	3,13 (1,81)	<0,001	5,8 (1,51)	3,6 (1,82)	3,26 (1,96)	<0,001*
Valor de p (grupos)					0,196			

Teste de Friedman para avaliar a diferença entre os diferentes momentos; teste de Mann-Whitney para avaliar a diferença entre os grupos no caso das variáveis com distribuição não normal. * Análise de variância com medidas repetidas para variáveis com distribuição normal (interação entre grupo e tempo); correções de Bonferroni ao nível de significância (nível de significância ajustado =0,05/número de comparações). RDAI: *Respiratory Distress Assessment Instrument.*

As comparações de RDAI ao longo do tempo mostraram que os dois grupos se comportaram de maneira similar (p=0,098) e que não houve diferenças entre os dois grupos (p=0,196). Os dois grupos apresentaram diminuição de RDAI 10 minutos após as intervenções (p<0,001) e mantiveram esses valores 60 minutos após. No Grupo 1, o escore passou de 5,02 para 3,06 após 10 minutos e para 3,13 após 60 minutos. Já no Grupo 2, o escore passou de 5,8 para 3,6 após 10 minutos e para 3,26 após 60 minutos (Figura 1 e Tabela 2).

**Figura 1:** Intervalos de confiança de 95% para os escores médios do *Respiratory Distress Assessment Instrument*, segundo o grupo e o tempo de avaliação

As respostas sobre os efeitos da fisioterapia foram satisfatórias na maioria dos itens avaliados pelo questionário utilizado, não havendo diferença entre os dois grupos (Tabela 3).

**Tabela 3:** Itens do questionário sobre a satisfação dos pais/responsáveis em relação às intervenções fisioterapêuticas realizadas

Itens do questionário	Grupo 1 n (%)	Grupo 2 n (%)	Valor de p
Criança tranquila durante as manobras de fisioterapia			
Não	1 (6,3)	0	>0,99
Sim	15 (93,8)	13 (100,0)	
Classificação das manobras de fisioterapia			
Relaxante	15 (100,0)	13 (100,0)	
Cansativa	0	0	
Manobras de fisioterapia ajudaram a melhorar a respiração da criança			
Não	1 (6,3)	0	>0,99
Sim	15 (93,8)	13 (100,0)	
Criança ficou tranquila durante a aspiração nasotraqueal			
Não	16 (100,0)	13 (100,0)	
Sim	0	0	
Aspiração nasotraqueal ajudou a melhorar a respiração da criança			
Não	0	0	
Sim	16 (100,0)	13 (100,0)	
Atendimentos de fisioterapia praticados por diferentes profissionais foram semelhantes			
Não	0	0	
Sim	16 (100,0)	13 (100,0)	
Criança foi beneficiada pelos atendimentos de fisioterapia			
Não	1 (6,3)	0	>0,99
Sim	15 (93,8)	13 (100,0)	
Melhora do sono			
Não	3 (18,8)	3 (23,1)	>0,99
Sim	13 (81,3)	10 (76,9)	
Melhora da dificuldade para respirar			
Não	0	1 (7,7)	0,448
Sim	16 (100,0)	12 (92,3)	
Melhora da alimentação			
Não	9 (56,3)	6 (46,2)	0,588
Sim	7 (43,8)	7 (53,8)	
Melhora do entupimento do nariz			
Não	2 (12,5)	2 (15,4)	>0,99
Sim	14 (87,5)	11 (84,6)	
Melhora do humor			
Não	9 (56,3)	5 (38,5)	0,340
Sim	7 (43,8)	8 (61,5)	
Realização de pesquisas como esta é importante			
Não	0	0	
Sim	16 (100,0)	13 (100,0)	
Recomendaria a fisioterapia respiratória para um parente ou amigo			
Não	1 (6,3)	0	>0,99
Sim	15 (93,8)	13 (100,0)	

Análise de variância com medidas repetidas para variáveis com distribuição normal (interação entre grupo e tempo); correções de Bonferroni ao nível de significância (nível de significância ajustado =0,05/número de comparações).

## DISCUSSÃO

O tratamento da bronquiolite geralmente é composto por terapêutica farmacológica, que objetiva atenuar o quadro fisiopatológico, e pela fisioterapia, com o objetivo de proporcionar higiene brônquica, otimização da reexpansão pulmonar e melhora da mecânica respiratória, com consequente prevenção de complicações pulmonares.^(14,15)^

Apesar da fisiopatologia da infecção ocasionada pelo VSR sugerir uma terapêutica efetiva com corticoide com ação anti-inflamatória no tratamento da bronquiolite, estudos não demonstram benefício da utilização dessa terapêutica.^(16)^ Segundo as Diretrizes Clínicas na Saúde Complementar da Sociedade Brasileira de Pediatria, o uso de corticosteroides não é recomendado no tratamento ambulatorial ou hospitalar da bronquiolite aguda (grau de recomendação A).^(17)^

A terapêutica com broncodilatadores é mais efetiva no estágio inicial da infecção, momento em que as pequenas vias aéreas não estão obstruídas com secreções.^(16)^ De acordo com as Diretrizes Clínicas na Saúde Complementar da Sociedade Brasileira de Pediatria, o uso rotineiro de broncodilatadores inalatórios não é recomendado no tratamento ambulatorial ou hospitalar para melhorar o prognóstico da bronquiolite aguda (grau de recomendação A).^(17)^

No estudo de Pinto et al*.*, cerca de 40% dos pacientes utilizaram antibiótico, sem diferenças entre os grupos, similarmente a estudo recente, randomizado, com 184 crianças menores que 12 meses de idade e hospitalizadas com bronquiolite. O tratamento com antibiótico não reduziu a duração da internação e nem a necessidade de oxigênio suplementar, concluindo que, na bronquiolite, o tratamento com antibióticos não deve ser utilizado rotineiramente, mas somente quando houver evidência de uma infecção bacteriana.^(16)^

A fisioterapia respiratória tem sido empregada rotineiramente em pacientes com bronquiolite, porém seus benefícios ainda são questionados, pela escassez de ensaios clínicos sobre o tema e pela limitada qualidade metodológica das pesquisas, que compromete qualquer afirmação que possa ser feita com relação a efeitos positivos da fisioterapia em pacientes com bronquiolite.^(18)^

Já em estudo recente, em que a fisioterapia foi realizada em todos os pacientes, foram evidenciadas menores necessidades de internação em UTI e de suporte ventilatório, em relação aos dados descritos na literatura.^(14)^

Alguns estudos relataram que a fisioterapia não deve ser indicada na fase aguda da bronquiolite, pelo fato de as manobras de higiene brônquica causarem agitação na criança, levando à hipoxemia e desencadeando broncoespasmo. Por outro lado, estudos relatam que a fisioterapia causa grande benefício nessas crianças, promovendo diminuição do tempo de internação hospitalar e evitando a necessidade de suporte ventilatório.^(6,19)^

A literatura compara ainda as diferentes intervenções, como tapotagem e vibração, associadas à drenagem postural, e não demonstra benefícios na evolução do quadro clínico da doença.^(8,20)^ Porém, estudos franceses demonstram benefícios na remoção da secreção pela técnica AFE, além das intervenções já citadas.^(21)^ A AFE potencializa a fisiologia pulmonar normal por meio das variações de fluxos aéreos, para a desobstrução brônquica e homogeneização da ventilação pulmonar.^(22)^

No estudo de Castro et al., no qual foram avaliados os efeitos da fisioterapia respiratória em pacientes com bronquiolite, não foram observadas diferenças estatisticamente significantes em relação à oxigenoterapia e à SpO_2_ antes e após os atendimentos.^(23)^ Neste estudo, em ambos os grupos, na maioria dos atendimentos, os pacientes apresentaram SpO_2_≥92% em todos os momentos observados, sem alterações significativas.

O escore RDAI apresenta boa confiabilidade na avaliação da presença e localização de sibilos e retrações, com pontuação de 1 a 17, de acordo com a intensidade dos sinais. Escores mais elevados indicam doença mais grave, sendo que uma pontuação <4 indica doença leve e uma pontuação >15, doença grave.^(13)^ No presente estudo, ambos os grupos se comportaram de maneira similar na avaliação do escore RDAI, sendo observada uma redução significativa do escore do momento pré para 10 minutos.

Durante a análise do questionário entregue aos responsáveis, foi observada redução imediata do desconforto respiratório após a fisioterapia respiratória, sendo esse benefício evidente. Apesar da escassez de evidências científicas sobre a repercussão da fisioterapia respiratória em pacientes com bronquiolite aguda, as técnicas de higiene brônquica têm sido solicitadas para o tratamento desses pacientes em diversos serviços, devido à melhora do quadro respiratório e à redução de complicações pulmonares, observadas na prática clínica. Tais técnicas são recomendadas em casos de obstrução brônquica devido à secreção.^ ( 18)^

Na aplicação diária de técnicas fisioterapêuticas, foi identificada uma redução mais rápida de sintomas respiratórios e clínicos, como diminuição da febre e dispneia, aumento do apetite, melhora da ausculta pulmonar e da tosse.^(24)^ No presente estudo, foram observadas respostas satisfatórias sobre os efeitos da fisioterapia na maioria dos itens avaliados pelos responsáveis. Todos os pacientes apresentaram melhora da respiração após a intervenção fisioterapêutica. Foi constatada pouca diferença entre os pacientes que apresentaram ou não melhora da alimentação e do humor, porém sem significância estatística.

Dentre as limitações deste estudo, podemos citar a dissimilaridade dos grupos, em relação ao uso de corticoide e internação na UTI, e as recusas por parte dos pais em relação à manobra AFE, devido à agitação e à irritabilidade observadas nas crianças submetidas a essa manobra.

## CONCLUSÃO

Não foram observadas diferenças entre os grupos em relação aos itens avaliados (tempo necessário para o paciente atingir alta do estudo, oximetria de pulso em ar ambiente e gravidade da doença pelo escore *Respiratory Distress Assessment Instrument*). A fisioterapia respiratória promoveu melhora do desconforto respiratório em pacientes com bronquiolite, principalmente após 10 minutos, e manutenção de oxigenação adequada em todos os momentos, sendo similar em ambos os grupos. Além disso, foram observadas respostas satisfatórias dos responsáveis sobre os efeitos da fisioterapia na maioria dos itens avaliados, tanto para a técnica aceleração do fluxo expiratório quanto para a tapotagem. Apesar dos resultados positivos deste estudo, faz-se necessária a realização de mais ensaios clínicos, a fim de avaliar e comparar a efetividade de diferentes técnicas fisioterapêuticas nesses pacientes.
